# Sorting through the Wealth of Options: Comparative Evaluation of Two Ultraviolet Disinfection Systems

**DOI:** 10.1371/journal.pone.0107444

**Published:** 2014-09-23

**Authors:** Michelle M. Nerandzic, Christopher W. Fisher, Curtis J. Donskey

**Affiliations:** 1 Research Service, Louis Stokes Cleveland Veterans Affairs Medical Center, Cleveland, Ohio, United States of America; 2 Geriatric Research, Education and Clinical Center, Cleveland Veterans Affairs Medical Center, Cleveland, Ohio, United States of America; 3 STERIS Corporation, Healthcare Group, Mentor, Ohio, United States of America; University of Connecticut, United States of America

## Abstract

**Background:**

Environmental surfaces play an important role in the transmission of healthcare-associated pathogens. Because environmental cleaning is often suboptimal, there is a growing demand for safe, rapid, and automated disinfection technologies, which has lead to a wealth of novel disinfection options available on the market. Specifically, automated ultraviolet-C (UV-C) devices have grown in number due to the documented efficacy of UV-C for reducing healthcare-acquired pathogens in hospital rooms. Here, we assessed and compared the impact of pathogen concentration, organic load, distance, and radiant dose on the killing efficacy of two analogous UV-C devices.

**Principal Findings:**

The devices performed equivalently for each impact factor assessed. Irradiation delivered for 41 minutes at 4 feet from the devices consistently reduced *C. difficile* spores by ∼ 3 log_10_CFU/cm^2^, MRSA by>4 log_10_CFU/cm^2^, and VRE by >5 log_10_CFU/cm^2^. Pathogen concentration did not significantly impact the killing efficacy of the devices. However, both a light and heavy organic load had a significant negative impacted on the killing efficacy of the devices. Additionally, increasing the distance to 10 feet from the devices reduced the killing efficacy to ≤3 log_10_CFU/cm^2^ for MRSA and VRE and <2 log_10_CFU/cm^2^ for *C.difficile* spores. Delivery of reduced timed doses of irradiation particularly impacted the ability of the devices to kill *C. difficile* spores. MRSA and VRE were reduced by >3 log_10_CFU/cm^2^ after only 10 minutes of irradiation, while *C. difficile* spores required 40 minutes of irradiation to achieve a similar reduction.

**Conclusions:**

The UV-C devices were equally effective for killing *C. difficile* spores, MRSA, and VRE. While neither device would be recommended as a stand-alone disinfection procedure, either device would be a useful adjunctive measure to routine cleaning in healthcare facilities.

## Introduction

Environmental surfaces may play an important role in transmission of healthcare-associated pathogens such as *Clostridium difficile*, methicillin-resistant *Staphylococcus aureus* (MRSA), and vancomycin-resistant *Enterococcus* (VRE) [1]–[6]. Pathogens are shed onto environmental surfaces and will remain for several days, or possibly months, if the surfaces are not effectively disinfected [1]–[6]. Unfortunately, several recent studies have demonstrated that environmental cleaning is often suboptimal in healthcare facilities [5]–[8]. Interventions such as education of housekeeping staff or use of fluorescent markers to provide feedback to housekeepers may result in improved cleaning [5]–[8]. Yet, despite the promise of improvement in routine cleaning, there remains a demand for novel, automated technologies that are effective against hard to kill *Clostridium difficile* spores, but are also safe and rapid. As a consequence, there has been an upsurge in automated disinfection technologies on the market, many of which have yet to be rigorously evaluated.

Novel ultraviolet disinfection devices are currently on the fore-front of burgeoning automated technologies due to the well documented efficacy of ultraviolet-C (UV-C) irradiation for killing bacteria, viruses, and persistent spores [9]–[15]. The mechanism of killing of microorganisms by UV-C is primarily due to inactivation of DNA and RNA through absorption of photons resulting in formation of pyrimidine dimers from thymine and cytosine [9], [12], [15]. We previously demonstrated that an automated room disinfection device that utilizes low pressure mercury lamps for emitting UV-C radiation was effective for significantly reducing *C. difficile*, MRSA, and VRE contamination in hospital rooms (Tru-D Rapid Room Disinfection, Lumalier, Memphis, TN, USA) [16]. Similarly, Rutala et al. evaluated the Tru-D device and concluded that it was an efficacious and environmentally friendly method for disinfecting surfaces in healthcare facilities [17]. Here, we performed a side-by-side comparative evaluation of a homologous automated UV-C room disinfection device (Pathogon UV Disinfection System, Steris Corporation, Mentor, Ohio, USA) against the previously tested Tru-D device, in the laboratory setting. For each device, the impact of pathogen concentration, organic load, distance, and radiant dose on killing efficacy was assessed.

## Materials and Methods

### *C. difficile*, MRSA, and VRE Strains

Two clinical isolates of C. difficile, MRSA and VRE were studied. The MRSA strains were a pulsed-field gel electrophoresis (PFGE) type USA300 (community-associated) and USA800 (hospital-associated). The VRE strains were a VanA-type isolate (C37) and a VanB-type isolate (C68). The *C. difficile* strains were VA 17, a restriction endonuclease analysis (REA) type BI strain, and VA 11, an REA type J strain.

### Preparation of *C. difficile* Spores

Spores were prepared by growth on brain-heart infusion agar (Becton Dickinson, Cockeysville, MD) supplemented with yeast extract (5 mg/ml) and L-cysteine (0.1%) at 37°C under anaerobic conditions as previously described [18]. Spores were stored at 4°C in sterile distilled water until use. Prior to testing, spore preps were confirmed by phase contrast microscopy and malachite green staining to be > 99% dormant, bright-phase spores.

### Microbiology

For VRE, MRSA, and *C. difficile* cultures, media included Enterococcosel agar (Becton Dickinson, Cockeysville, MD) containing 20 µg/mL of vancomycin, CHROMagar (CHROMagar, Paris, France) containing 6 µg/mL of cefoxitin, and cycloserine-cefoxitin-brucella agar containing 0.1% taurocholic acid and lysozyme 5 mg/L (CDBA), respectively [19]. Plates containing MRSA or VRE were incubated aerobically at 37°C for 48 hours. *C. difficile* plates were incubated in a Whitley Workstation MG1000 anaerobic chamber (Microbiology International, Frederick, MD) at 37°C for 48 hours.

### The UV-C Disinfection Devices

**Figure 1:**
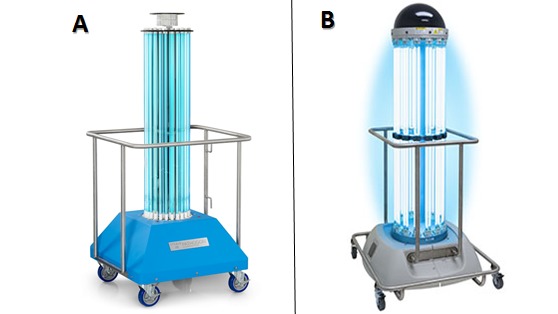
Photographs of the Pathogon (A) and Tru-D (B) devices.

Figure 1A/B is a photograph of the devices. The Pathogon device (1A) is 28 inches wide, 31 inches long, and stands 67 inches tall. The system is a wheeled mobile unit that is controlled remotely by a Windows-based tablet controller. It is placed in the center of the room and commonly touched surfaces are arranged close to the device for optimal exposure to UV-C radiation (i.e. bedrails pulled up, call buttons placed on the bed, tables placed near the device). The device contains motion and heat sensor that are connected to a safety rated relay, aborting the UV-C cycle if someone enters the room during use. The unit has 24, 45 inch low pressure mercury bulbs. Once the operator has exited the room, a pre-programed germicidal dose is chosen based on the dimensions of the room. UV-C radiation penetrates all areas of the room that receive light, but the highest exposure occurs for areas that are in direct line of exposure to the output of the device; areas that are not in direct line of exposure to UV-C may receive radiation that is reflected from the walls and ceiling or from other surfaces in the room.

The Tru-D device (1B) is 72 inches tall and measures 24 inches at the widest portion of the base. It is a wheeled mobile device that is placed strategically in the center of the room just as described above for the Pathogon device. The operator exits the room, closes the door, and places a door sensor on the frame of the door. Continuous monitoring during operation is not required because the sensor triggers automatic discontinuation of the cycle if the door is opened. A handheld remote is used to select either a vegetative cycle that is effective for killing of non-spore forming organisms or a spore cycle that is effective in killing spores. The unit has 28, 36 inch low pressure mercury bulbs. The device contains eight sensors spaced at equal distances on a ring at the top of the device. The sensors measure the amount of UV-C light reflected back to the device. The device automatically ends the cycle when the area reflecting the lowest level of UV-C back to the sensors (i.e. shaded areas in the room) has received an adequate dose.

### Efficacy of the UV-C Devices for the Reduction of Pathogens on Carriers

#### The Effect of Pathogen Concentration and Organic Load

Initial experiments were conducted to determine whether pathogen concentration (i.e. concentration of organisms per cm^2^) or organic load influenced the disinfection efficacy of the UV-C devices. For pathogen concentration experiments, ten µl aliquots of two strains of *C. difficile* spores, MRSA, and VRE were suspended in sterile phosphate buffered saline (PBS), inoculated onto stainless steel carriers, and then spread to cover a 1 cm^2^ area. Organisms were allowed to desiccate onto the carriers under ambient room conditions. For *C. difficile* spores, the inoculum applied to the carriers ranged from 2 to >5 log_10_CFU/cm^2^. Previous experiments demonstrated that a reduction in vegetative organisms (MRSA and VRE) was observed after initial desiccation onto the carriers, however no further reduction was observed within the duration of treatment time (author's unpublished data). For each vegetative pathogen, the inoculum applied to the slide was adjusted such that 2 to >5 log_10_CFU/cm^2^ were recovered from the positive control specimens after desiccation. For organic load experiments, two strains of *C. difficile* spores, MRSA, and VRE were suspended in either sterile PBS, light organic load (5% fetal calf serum), or heavy organic load (5% fetal calf serum and 5% tryptone) and inoculated onto stainless steel carriers as described above. However, the inoculum was altered such that each carrier yielded 6 log_10_ CFU at baseline.

The carriers were placed on a laboratory bench top 4 feet from the UV-C device, within the direct field of radiation. Baseline slides were left untreated outside of the room (i.e., positive controls). The room dimensions were approximately 10×10 feet. Based on these dimensions, the UV-C devices were run for 41 minutes, as suggested by the manufacturer of the Pathogon device to deliver a spore-killing dose of UV-C within a 10×10 foot range.

To quantify viable organisms, the carriers were submersed in 10 mL of sterile PBS, vortexed vigorously, and dilutions of the suspensions were plated onto selective media as described in *Microbiology*. Following 48 hours of incubation, log_10_ colony forming unit (CFU) reductions were calculated by comparing the log_10_CFU recovered from carriers post UV-C disinfection to untreated controls. All experiments were repeated three times.

#### The Effect of Distance and Indirect Irradiation

The killing efficacies of the UV-C devices were evaluated at increasing distances and shaded from the direct field of radiation. Carriers were prepared and processed as described above in *The Effect of Pathogen Concentration and Organic Load*, however, the organisms were suspended in PBS and altered such that each carrier yielded 6 log_10_ CFU at baseline. Additionally, carriers were placed 6 inches, 4 feet and 10 feet within the direct field of radiation, and also 4 feet shaded from direct radiation. The UV-C devices were run for 41 minutes.

#### The Effect of Radiant Dose

The effect of radiant dose on the killing efficacies of the UV-C devices was determined for vegetative organism (MRSA and VRE) and spores (*C. difficile*). Carriers were prepared and processed as described above in *The Effect of Pathogen Concentration and Organic Load*, however, the organism were suspended in PBS and altered such that each carrier yielded 6 log_10_ CFU at baseline. Carriers were placed 4 feet from the device in the direct field of UV-C and irradiated for either 10, 20, or 40 minutes.

### Statistical Analysis

Data were analyzed using STATA 9.0 software (StataCorp, College Station, TX). Continuous data were analyzed using paired *t* tests.

## Results

### Efficacy of the UV-C Devices for the Reduction of Pathogens on Carriers

#### The Effect of Pathogen Concentration and Organic Load

**Figure 2:**
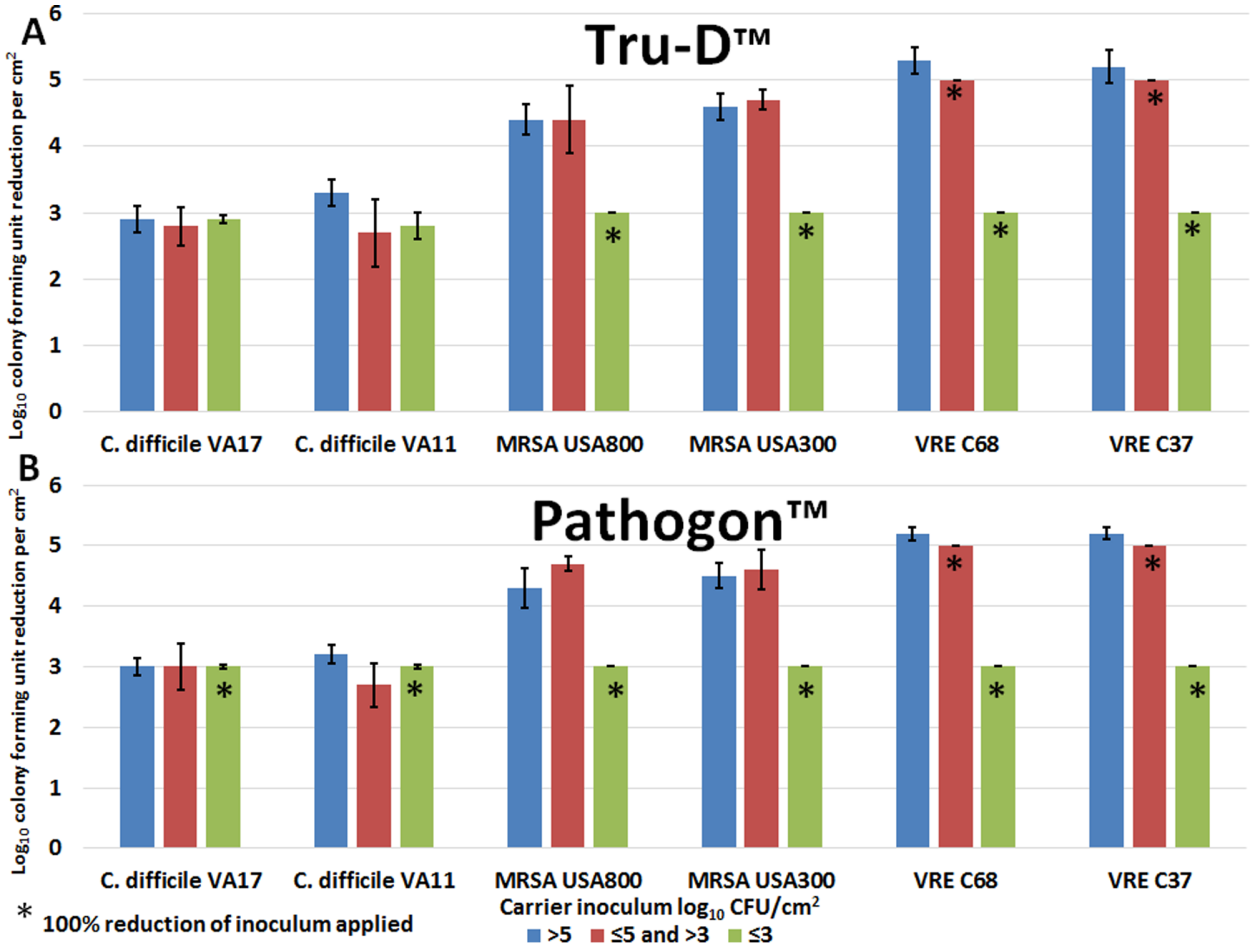
The effect of pathogen concentration on the efficacy of the UV-C devices. The log_10_CFU reduction/cm^2^ of two strains of *C. difficile* spores, MRSA, and VRE inoculated onto carriers. Carriers contained either >5, ≤5 and >3, or ≤3 log_10_CFU of each pathogen. The carriers were irradiated for 41 minutes at a distance of 4 feet from the Tru-D (2A) or Pathogon (2B) device. The means of the data from experiments conducted in triplicate are presented. Error bars indicate standard error.

Figures 2A and 2B show the mean log_10_CFU/cm^2^ reductions of two strains of *C. difficile*, MRSA, and VRE on carriers after the use of the Tru-D and Pathogon devices, respectively. There was no significant differences between the log_10_CFU reductions of the two strains of each pathogen tested. Therefore, in subsequent experiments, the two strains were calculated collectively in the mean. The concentration of pathogens on a surface (≤3 to>5 log_10_CFU) did not have an impact on the killing efficacy of the UV-C devices. Furthermore, the UV-C devices were equally effective for reducing pathogens. There was no significant difference observed in the log_10_CFU reductions achieved by the Tru-D or Pathogon device for the two strains of each pathogen assessed (Tru-D vs. Pathogon: *P* = 0.57 (*C.difficile*), *P* = 1.0 (MRSA) and *P* = 0.97 (VRE)). Irradiation delivered by the Tru-D and Pathogon devices for 41 minutes (spore killing dose) consistently reduced *C. difficile* spores by ∼ 3 log_10_CFU/cm^2^, MRSA by >4 log_10_CFU/cm^2^, and VRE by >5 log_10_CFU/cm^2^.

**Figure 3:**
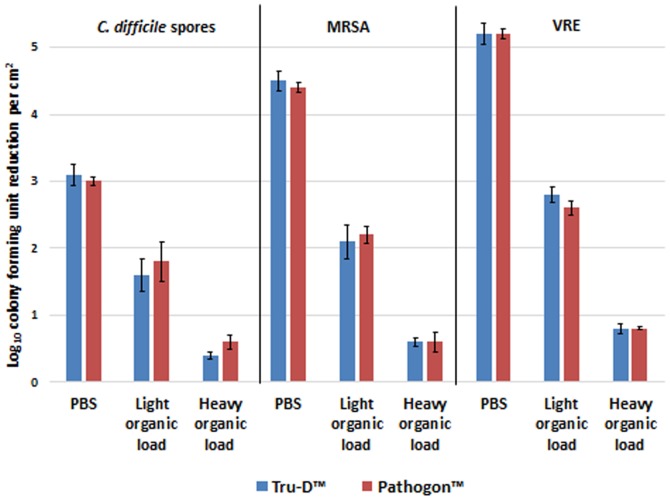
The effect of organic load on the efficacy of the UV-C devices. The log_10_CFU reduction/cm^2^ of *C. difficile* spores, MRSA, and VRE suspended in phosphate-buffered saline (PBS), light organic load (5% fetal calf serum), or heavy organic load (5% fetal calf serum, 5% tryptone). Carriers contained 6 log_10_CFU of each pathogen. The carriers were irradiated for 41 minutes at a distance of 4 feet from the Tru-D or Pathogon device. The means of the data from experiments conducted in triplicate are presented. Error bars indicate standard error.

Figure 3 shows the effects of a light and heavy organic load on the killing efficacy of the Tru-D and Pathogon device. Both the light (5% fetal calf serum) and heavy organic load (5% fetal calf serum, 5% tryptone) had a significant deleterious impact on the efficacy of the devices. The light organic load decreased the log reductions achieved by the devices to <2 log_10_CFU/cm^2^ for *C.difficile* spores, <2.5 log_10_CFU/cm^2^ for MRSA, and <3 log_10_CFU/cm^2^ for VRE. The heavy organic load had a more dramatic effect, decreasing the log reduction to <1 log_10_CFU/cm^2^ for each pathogen assessed.

#### The Effect of Distance and Indirect Irradiation

**Figure 4:**
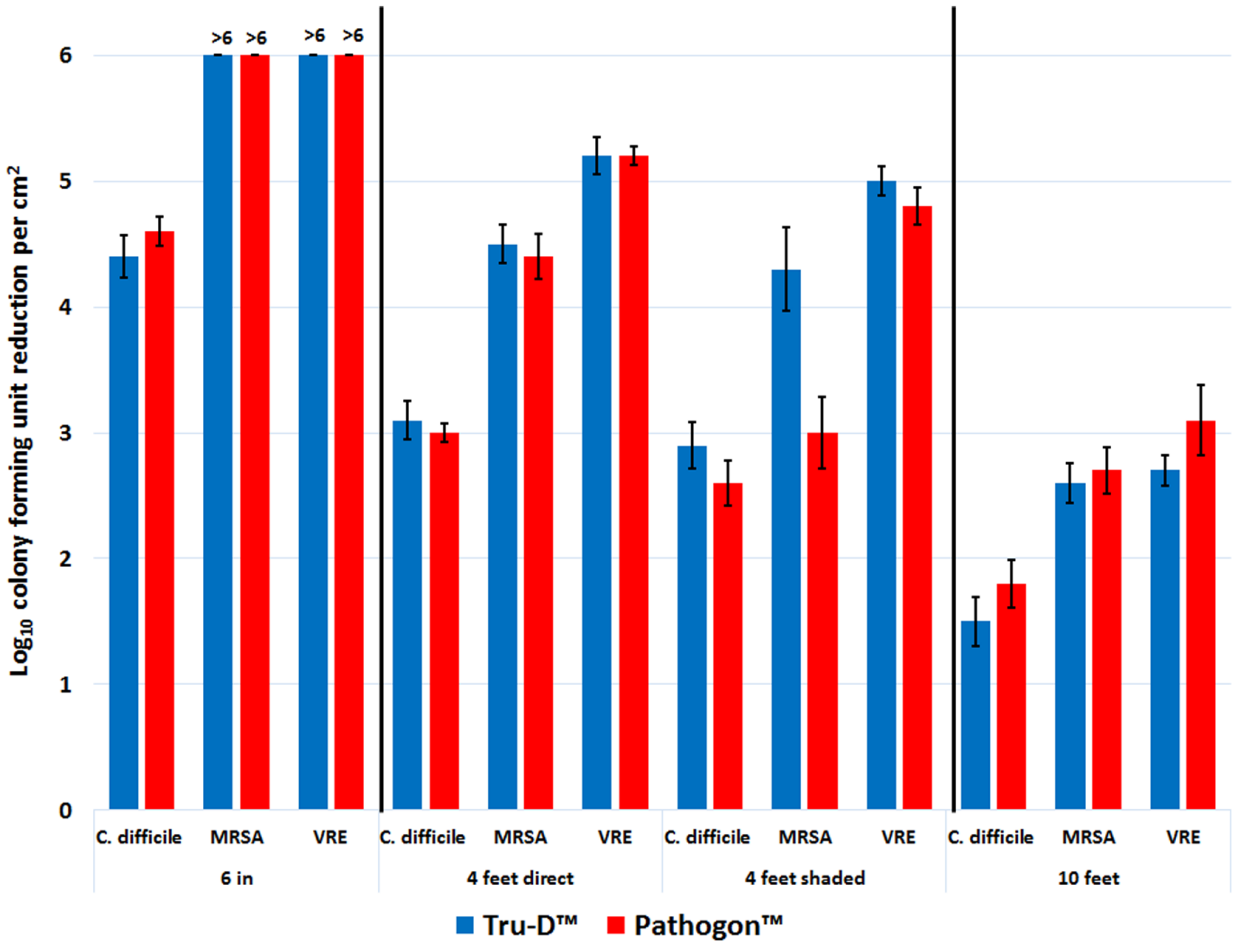
The effect of distance and indirect irradiation on the efficacy of the UV-C devices. The log_10_CFU reduction/cm^2^ of *C.difficile* spores, MRSA, and VRE at increasing distances and shaded from the direct field of radiation delivered by the UV-C device. Carriers contained 6 log_10_CFU of each pathogen. The carriers were irradiated for 41 minutes at a distance of 6 in, 4 feet, 4 feet shaded, and 10 ft from the Tru-D or Pathogon device. The means of the data from experiments conducted in triplicate are presented. Error bars indicate standard error.

The germicidal efficacy of UV-C light as a function of distance follows an inverse relationship, as shown in Figure 4. The Tru-D and Pathogon devices achieved analogous log reductions for each distance assessed. Six inches away from the device, vegetative organisms (MRSA and VRE) were completely eliminated (≥6 log_10_CFU/cm^2^) and *C. difficile* spores were reduced by >4 log_10_CFU/cm^2^. As the distance from the device was increased to 4 feet, the log reduction decreased to ≤5 log_10_CFU/cm^2^ for vegetative organisms and ≤3 log_10_CFU/cm^2^ for *C. difficile* spores. Shading the organisms from the direct field of radiation did not have a significant impact on the killing efficacy of the devices. Ten feet from the devices, the log reductions decreased further to ≤3 log_10_CFU/cm^2^ for vegetative organisms and <2 log_10_CFU/cm^2^ for *C.difficile* spores.

#### The Effect of Radiant Dose

**Figure 5:**
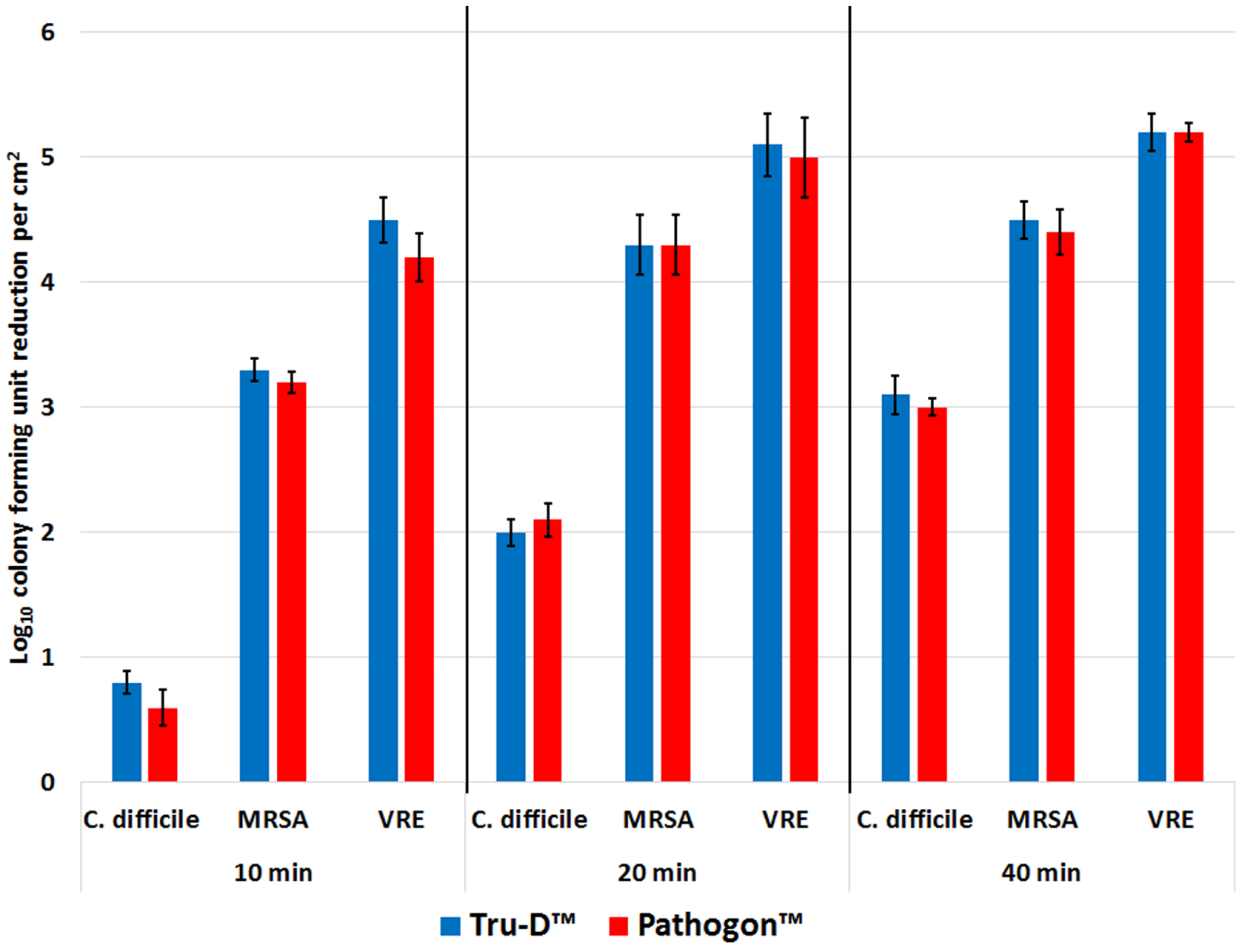
The effect of radiant dose on the efficacy of the UV-C devices. The log_10_CFU reduction/cm^2^ of *C.difficile* spores, MRSA, and VRE after receiving increasing timed doses of irradiation delivered by the UV-C devices. Carriers contained 6 log_10_CFU of each pathogen. The carriers were irradiated for 10, 20, 40 minutes at a distance of 4 feet from the Tru-D or Pathogon device. The means of the data from experiments conducted in triplicate are presented. Error bars indicate standard error.

Figure 5 shows the effect of radiant dose on the killing efficacy of the UV-C devices. There was no significant difference between the killing efficacies of the Tru-D or Pathogon device for each of the timed doses of irradiation delivered. Killing achieved by the UV-C devices was directly proportional to the dose of irradiation delivered. MRSA and VRE were reduced by >3 log_10_CFU/cm^2^ after only 10 minutes of irradiation, while the hardier *C. difficile* spores required 40 minutes of irradiation to achieve a >3 log_10_CFU/cm^2^ reduction.

## Discussion

We found that the Tru-D and Pathogon devices were equally effective for killing *C. difficile* spores, MRSA, and VRE in a laboratory setting. Surfaces in a real-world setting contain variable levels of contamination, and in our experience, yield between 4 to <1 log_10_CFU when cultured (author's unpublished data). Here, we determined that the concentration of pathogens on a surface did not have a significant impact on the killing efficacy of the UV-C devices. Conversely, organic load did significantly reduce the killing efficacy of both devices. These findings are inconsistent with previously published data showing that organic load did not impact the killing efficacy of the Tru-D device [16]. However, the organic load used in the current study was much more concentrated (5% fetal calf serum or 5% fetal calf serum plus 5% tryptone) than in the previously published study (1% bovine serum albumin). The current study demonstrates that as the matrix of organic load increased from light to heavy, the killing efficacy of the devices decreased, suggesting that UV-C light does not penetrate heavy soils, but may break through lighter organic loads.

Other factors known to impact the delivery of lethal doses of UV-C irradiation are distance from the device and time of radiant exposure [16]–[17]. The efficacy of the Tru-D and Pathogon devices significantly decreased as distance from the devices increased. We have previously demonstrated that shading from the direct field of irradiation inhibited the lethal effects of the Tru-D device (assessed at 10 feet from the device) [16]. Conversely, here we found that at 4 feet from the devices, shading did not have a significant impact on the killing efficacy of UV-C. For killing of *C. difficile* spores, time of UV-C exposure was of particular importance. While vegetative organisms were reduced by 3 log_10_CFU after only 10 minutes of exposure, it took 40 minutes to achieve the same level of reduction for *C. difficile* spores. These results suggest that the Tru-D and Pathogon devices are similarly effective at delivering lethal doses of UV-C irradiation under analogous conditions. And as previously demonstrated for the Tru-D device, the Pathogon device may be a promising new environmental disinfection technology that could be a useful adjunct to routine cleaning measures in healthcare facilities.

UV-C devices have important advantages over other disinfection strategies that are effective against *C. difficile* spores. Sodium hypochlorite has corrosive effects on various materials, may irritate the eyes and respiratory tracts of cleaning staff and patients, and the efficacy is dependent on correct application by housekeeping staff [20]. Hydrogen peroxide vapor and hydrogen peroxide dry-mist have been shown to be highly effective in elimination of *C. difficile* spores [20]–[22]. However, these systems are relatively expensive to operate, a dedicated staff is required, and up to several hours may be required to complete room disinfection [20]–[22]. In contrast, after the initial purchase of the UV-C device, the cost of operating and maintaining them is minimal (i.e., electricity and annual bulb replacement of ∼ $20 each), a dedicated staff is not essential, and a 3 log_10_CFU reduction in *C. difficile* spores can be achieved in less than an hour. Additionally, UV-C may be less damaging to surfaces than bleach and does not produce emissions that are harmful or irritating to operators.

The Tru-D and Pathogon devices do have some potential limitations. First, because spores require a minimum of 40 minutes of irradiation to achieve significant reductions, it may not be feasible to use the devices in circumstances where rapid turn-over of rooms is required. Second, surface properties and organic debris may potentially inhibit lethal doses of UV-C from killing pathogens. For example, UV-C does not penetrate porous surfaces such as sheets, upholstery and curtains [15]. In our current study, lethal doses of UV-C irradiation were significantly or completely inhibited by organic matrices. Finally, the efficacy of the UV-C devices was reduced at sites further from the devices. Therefore, it is recommended that commonly touched surfaces (e.g., bedside table, call button, telephone) be arranged close to the device for optimal exposure to UV-C radiation.

Both UV-C disinfection systems performed similarly in a laboratory setting, however, each system has certain advantages and limitations. The Tru-D device is unique in that it uses UV-C light reflected from the walls, ceilings, floors, and items in the room to calculate the amount of irradiation required to deliver a programmed lethal dose for either vegetative or spore-forming pathogens. This feature is advantageous because it delivers a customized dose of irradiation to each room based on the areas of the room that are hardest for light to penetrate. However, this advantage translates into longer cycle times for rooms that inhibit the reflection of light, often increasing the run time to greater than 50 minutes for a 10×10 foot room. On the other hand, the Pathogon device delivers a pre-programmed dose of irradiation that is configured based on the size of the room and type of pathogen contamination suspected, therefore, cycle time does not fluctuate. However, shaded areas of the room may not receive sufficient lethal doses of irradiation. In our opinion, the Pathogon control interface is more user-friendly, however, the next generation of the Tru-D device has been updated with an iPad interface. Last, the Pathogon device is significantly less expensive than the Tru-D device, however after the initial cost, both units require equivalent care and maintenance.

Our study does have some limitations. This study was not designed to address the impact of the UV-C devices on native pathogens found on surfaces in hospital rooms. Nevertheless, the two devices performed equivalently in a laboratory setting, and because the Tru-D device has been previously reported as effective for significantly reducing *C. difficile*, MRSA, and VRE contamination in hospital rooms it can be inferred that the Pathogon device may perform similarly. Further studies are needed to determine whether reductions achieved by these devices translates to reduced rates of infection.
